# Efficacy of a reduced brace wear regimen supported by Schroth exercise in adolescent idiopathic scoliosis: a randomized trial

**DOI:** 10.3389/fped.2026.1774281

**Published:** 2026-04-01

**Authors:** Fanyuan Meng, Kerong Li, Moxian Chen, Zhi Zhao, Wei Wang, Lijuan Ao

**Affiliations:** 1 Department of Physical Therapy, The School of Rehabilitation, Kunming Medical University, Kunming, China; 2 Department of Rehabilitation, Kunming Municipal Hospital of Traditional Chinese Medicine, Kunming, China; 3 Department of Orthopaedics, The Second Affiliated Hospital of Kunming Medical University, Kunming, China; 4 College of Mechanical and Electrical Engineering, Harbin Engineering University, Harbin, China

**Keywords:** adolescent idiopathic scoliosis, full-time brace, part-time brace, quality of life, Schroth exercise

## Abstract

**Objectives:**

This study aimed to determine whether reducing daily brace wear duration, when compensated by intensive Schroth exercise, yields non-inferior long-term curve correction and superior quality of life compared to standard full-time bracing in AIS.

**Methods:**

A total of 52 AIS patients (Cobb angle 25°–40°) were randomly and equally assigned to a control group (full-time brace wear, 20–24 h/day) and an experimental group (part-time brace wear, 14–18 h/day combined with Schroth exercise). Specifically, the Schroth three-dimensional exercise including home-based and supervised outpatient sessions, were performed at least 5 times per week, with a minimum total duration of 4–5 h per week. Evaluations of Cobb angle, angle of trunk rotation (ATR), thoracic expansion, and the Scoliosis Research Society 22-item (SRS-22) questionnaire were conducted baseline and after 6 months of treatment. A subsequent follow-up assessment at 12-months was carried out, with the Cobb angle employed as the primary outcome to evaluate the long-term corrective efficacy between the two groups.

**Results:**

The two-way repeated measures ANOVA revealed a significant Time × Group interaction for the primary outcome, the Cobb angle (*P* < 0.001). At both the 6- and 12-month follow-ups, the Cobb angle was significantly reduced from baseline in both groups (*P* < 0.01), with the experimental group demonstrating superior improvement compared to the control group (*P* < 0.01). Regarding secondary outcomes at 6 months, the experimental group also showed significantly greater improvement than the control group in the ATR, thoracic expansion, and the pain and mental health domains of the SRS-22 (*P* < 0.05). Furthermore, within the experimental group, ATR, thoracic expansion, and the pain and mental health domains of the SRS-22 improved significantly from baseline at 6 months (*P* < 0.01), whereas in the control group, only the Cobb angle and ATR showed significant improvement (*P* < 0.01).

**Conclusions:**

This short-term study provides evidence that a protocol prioritizing intensive Schroth exercise combined with part-time bracing achieved superior corrective efficacy compared to full-time bracing alone for adolescent idiopathic scoliosis. These findings support integrating structured Schroth therapy as a core component within a part-time bracing regimen, suggesting a potential to reduce reliance on full-time wear while maintaining efficacy in the short term.

**Clinical Trial Registration:**

https://www.chictr.org.cn/showproj.html?proj=201861, identifier ChiCTR2300075910.

## Introduction

Adolescent idiopathic scoliosis (AIS) represents the most prevalent type of scoliosis. It is characterized by a three-dimensional spinal deformity, including lateral curvature, anteroposterior deviation, and axial rotation without an identifiable cause (1). AIS typically develops in children after age 10 (2), with an overall prevalence of 3% in the adolescent population and a strong female predominance (female-to-male ratio of 7:1) (3). Currently, the treatment strategies for AIS involve conservative and surgical treatment, guided by curve severity (measured by Cobb angle) and skeletal maturity (Risser sign). While spinal fusion remains the primary surgical option, it can cause considerable risks including chronic lower back pain, and temporary pulmonary function decline lasting up to 2 years postoperatively (4). Conservative treatment is therefore recommended for early-stage AIS to avoid surgery. The International Scientific Society on Scoliosis Orthopedic and Rehabilitation Treatment (SOSORT) guidelines (5) suggest that the conservative treatment aims to prevent curve progression during adolescence, address respiratory dysfunction, manage spinal pain, and optimize postural aesthetics.

The efficacy of bracing in controlling scoliosis progression is well-documented (6), with SOSORT guidelines recommending either part-time (12–20 h) and full-time rigid bracing (20–24 h) protocols. Research indicates that the effect of bracing is dose-dependent: optimal therapeutic effects are achieved with more than 22 h daily wear, clinically significant benefits are observed with 14–22 h, while wear time less than 14 h demonstrates negligible therapeutic effect (7). Patient compliance with prescribed wear time is crucial for treatment success, common barriers to adherence include aesthetic concerns, functional discomfort at pressure points, heat intolerance, and limited physical activity (8). In addition, the psychological impact of long-term bracing during adolescence cannot be ignored (9). Physiotherapeutic scoliosis-specific exercises (PSSE) offers individualized therapeutic protocols based on the curve-specific parameters including location, magnitude, and clinical presentation. among various PSSE protocols. The Schroth three-dimensional (3D) exercise, established by Ms. Katharina Schroth in 1921, was the most commonly used. This approach integrates motor-sensory training, postural education, and breathing techniques, emphasizing active self-correction of scoliotic deformities (10). However, limited evidence suggested that the Schroth approach independently or in combination with other conservative treatments can improve curve angles and the quality of life of patients with AIS (11).

Negrini et al. (12) demonstrated that bracing effectively reduces curve progression and prevents surgical intervention in AIS patients, with enhanced outcomes when combined with exercises according to the SOSORT criteria. However, current literature lacks evidence regarding the potential for Schroth exercise to achieve equivalent outcomes with reduced brace wear time. Additionally, the sustained therapeutic outcomes of Schroth 3D exercise intervention in mild-to-moderate AIS cases has not been definitively established (11). This study aimed to compare the short-term therapeutic efficacy of a combined regimen of part-time bracing and intensive Schroth therapy with that of conventional full-time bracing alone in adolescents with idiopathic scoliosis. We hypothesized that a combined regimen of intensive Schroth exercises and part-time bracing would yield superior clinical outcomes compared to a conventional full-time bracing protocol alone.

## Methods

### Study design and the participants

The clinical trial complied with the clinical trial guidelines (Consolidated Standards of Reporting Trials, CONSORT) and was approved by the Ethics committee of Kunming Medical University (approval number: KMMU2023MEC152). The protocol was registered with the Chinese Clinical Trial Registry (ChiCTR2300075910). All participants signed a statement of informed consent before participating in the study. From October 2023 to February 2024, 52 AIS patients aged over 10 years were recruited from the Orthopedics Department of the Second Affiliated Hospital of Kunming Medical University. Following enrollment, patients were randomly assigned to the control group (full-time brace wearing for 20–24 h/day) or the experimental group (part-time brace wearing for 14–18 h/day with Schroth exercise). The study employed a double-blind design. All patients underwent standardized outpatient medical evaluation including radiographic evaluation, trunk rotation measurements, thoracic expansion testing, Scoliosis Research Society 22-item (SRS-22) questionnaire administration.

The inclusion criteria of the patients were: (1) patients with AIS diagnosis; (2) female; (3) aged over 10 years; (4) Risser's sign 0-III; (5) primary Cobb angle 25°–40°; (6) no prior surgical treatment; and (7) patients who signed an informed consent form before the trial, acknowledging potential treatment-related discomfort and the option to voluntarily terminate the study. Exclusion criteria were (1) patients diagnosed with non-idiopathic scoliosis (congenital scoliosis, postural scoliosis, neuromuscular scoliosis, etc.); (2) patients with other comorbid cardiopulmonary diseases; and (3) patients with significant cognitive deficits affecting questionnaire completion. All patients provided written informed consent for blinded management of their clinical data.

**Figure 1:**
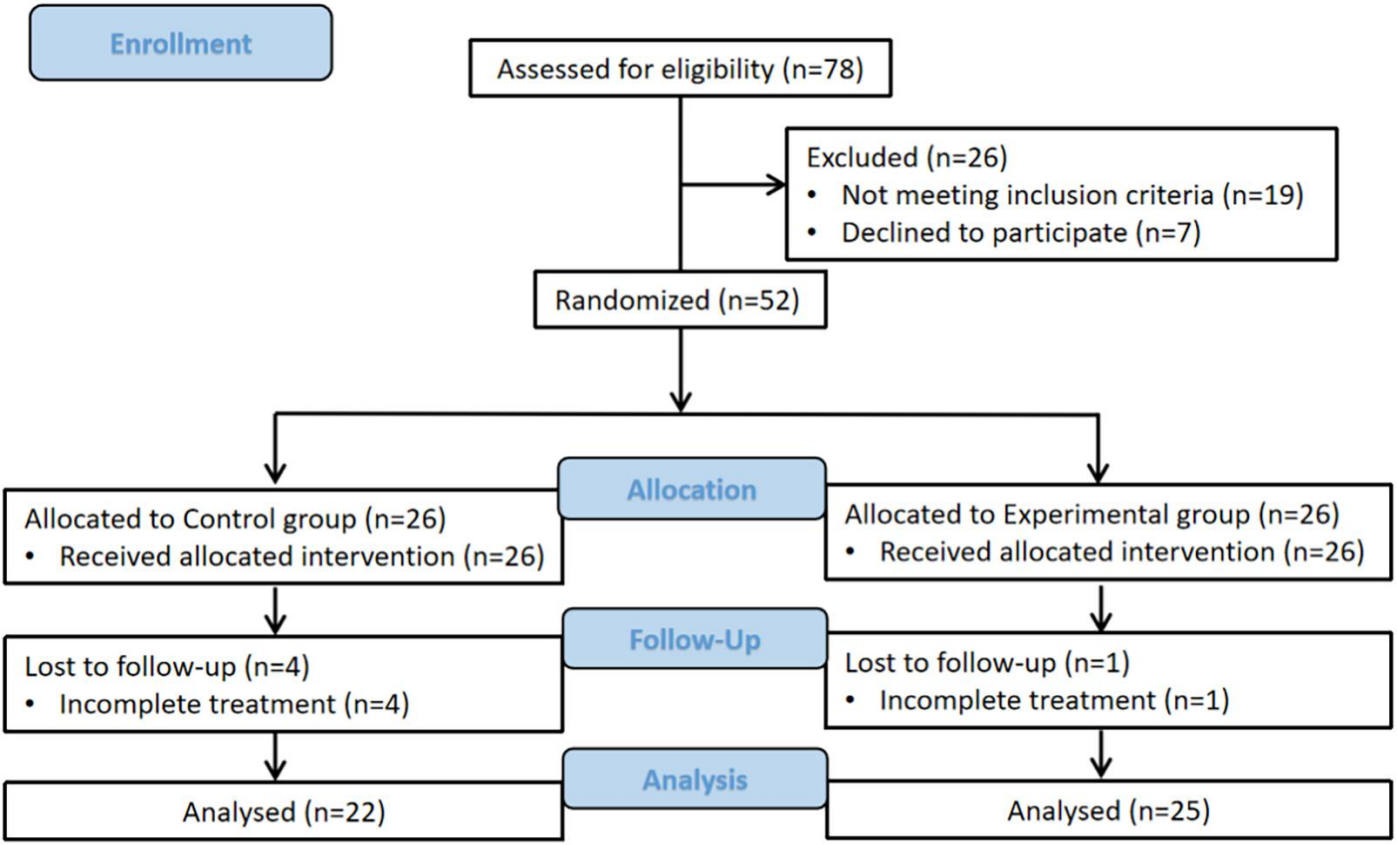
The CONSORT flow diagram of this study.

Sample size was calculated using G-Power software 3.1.9.7 (http://www.gpower.hhu.de). (Based on previously reported effect size (*f* = 0.25) from Yagci et al.'s reported (13). With statistical power (1−*β*) = 0.95, *α* = 0.05, 44 participants were required. Considering the drop-out rate of 20%, a total of 52 participants (26 per group) were recruited into the study. Initially, a total of 78 patients with AIS were recruited in this study. 26 patients (19 ineligible, 7 early dropouts) were excluded. Of the 19 patients excluded from analysis, most had received prior conservative treatment (bracing or PSSE) before enrollment. Separately, the 7 patients who withdrew from the study did so primarily due to scheduling conflicts and transportation difficulties that prevented consistent attendance at outpatient Schroth therapy sessions. The remaining 52 patients were randomized equally between groups. 4 patients in the control group and 1 patient in the experimental group withdraw, leaving 22 in the control group and 25 in the experimental group for final analysis. This information accompanies a CONSORT flow diagram (Figure 1).

According to Rigo's classification criteria, whole spine characteristics by radiographic evaluation (14) classified as four primary scoliosis patterns: 3C (major thoracic with or without a minor lumbar curve), 4C (major lumbar curve with a minor thoracic), N3N4 (double, well-balanced curves) and single lumbar curves. These patterns were further categorized into two groups: single curves (including single lumbar, single thoracolumbar, and 3C without a minor lumbar curve) and double curves (comprising 3C with a minor lumbar curve, 4C, and N3N4). Patients in the full-time brace group had 5 single curves and 17 double curves, while those in the part-time brace plus Schroth exercise group included 5 single curves and 20 double curves.

### Randomization and blinding

Fifty-two AIS patients were randomly divided into two groups: a full-time brace group and a combined treatment group. Matched pairs randomization was performed using the Research Randomizer program (randomizer.org) with matching criteria including Cobb angle, age, BMI, and Risser sign. This study employed a double-blind design in which both the participants and the outcome assessors were blinded to group allocation. All assessments were evaluated by the same physiotherapist (PT) at baseline, 6 months, and 12 months post-treatment initation.

### Interventions

Participants in the experimental group (part-time brace plus Schroth exercise) received combined treatment consisting of Schroth 3D exercise and orthotic intervention. Initial training involving 4–5 outpatient sessions conducted by the same certified Schroth PT, who ensured participants and their caregivers could accurately perform the exercises at home or school. Weekly outpatient sessions with the same PT continued throughout the study period, during which the therapist monitored exercise performance, adjusted the training program based on individual competency and completion quality. The Schroth exercises program involves 4–5 weekly sessions, each lasting 40–60 min, totaling 4–5 h per week for an initial 6-month period. Following this supervised clinical training, participants continued with a structured home exercise regimen. Treatment adherence was monitored by parent-signed checklists regarding to exercise completion and brace wear, and video recordings of home exercises. The therapist send out weekly training log sheets where patients recorded their daily activities, which were then submitted for weekly review.

**Figure 2:**
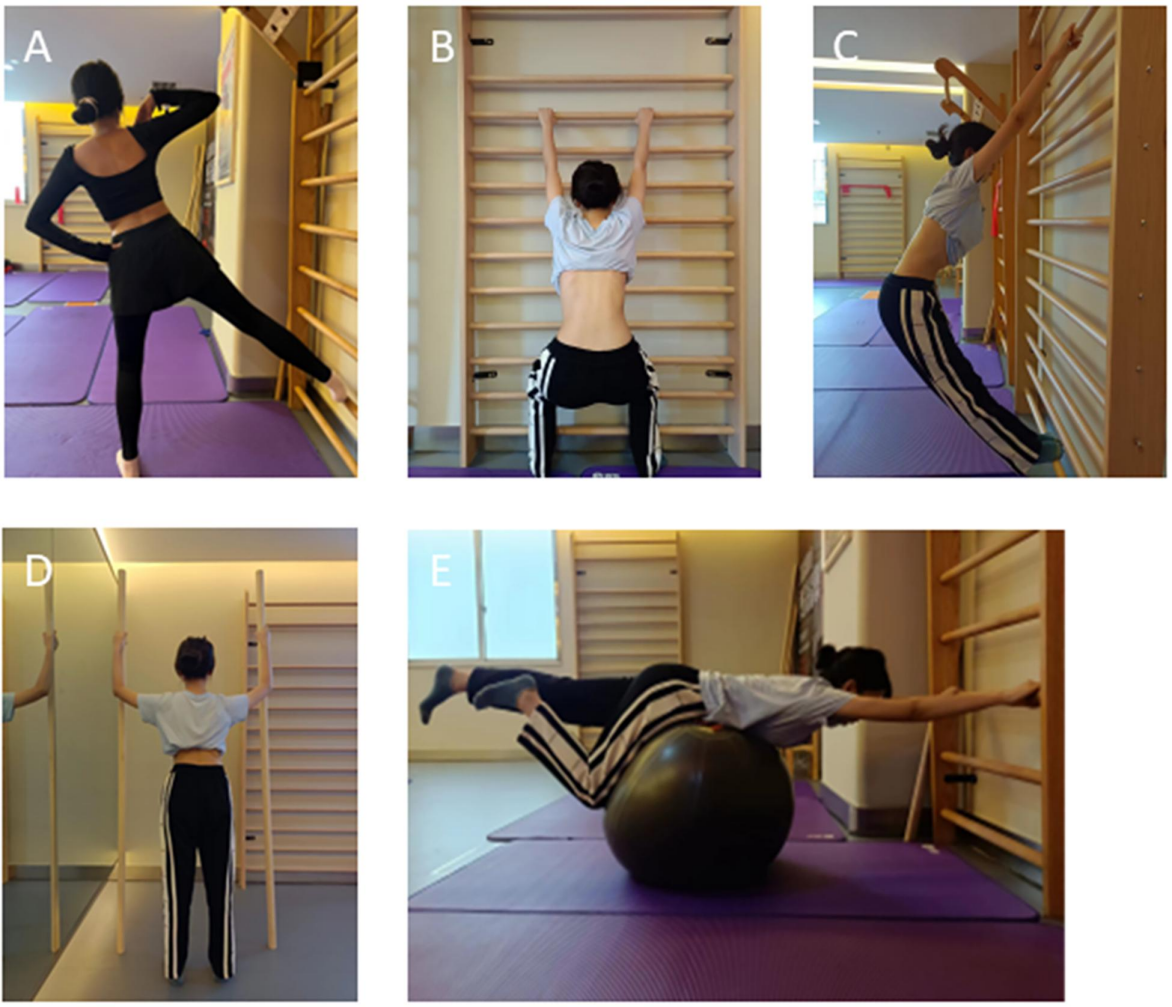
Schroth exercises demonstrated on a patient with Rigo 4C curve type. **(A)** Muscle cylinder exrecise, **(B)** Short semihanging position, **(C)** Big bow maneuver, **(D)** Standing correction between two Poles, **(E)** Hip flexion exercise on a stability ball.

**Figure 3:**
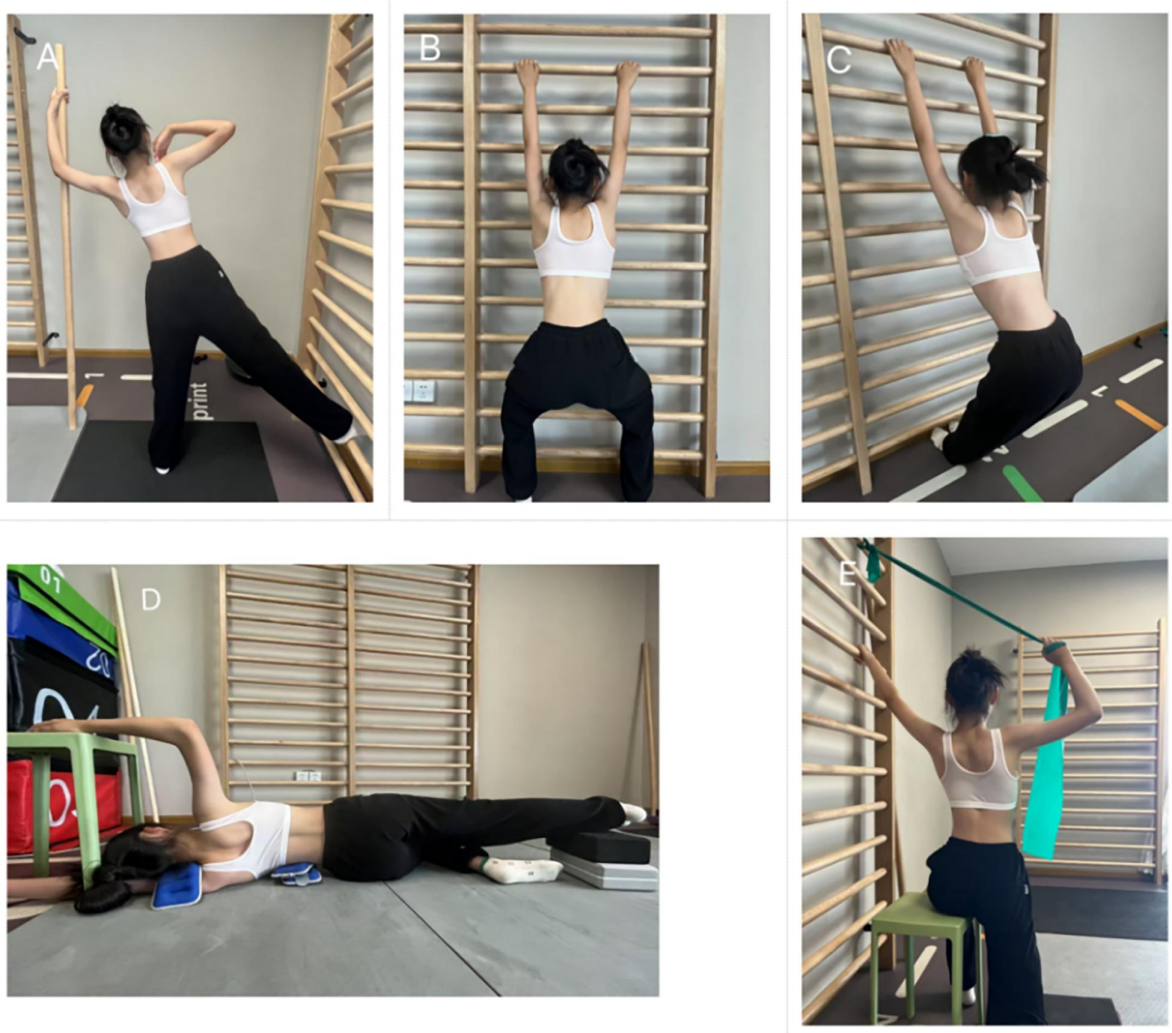
Schroth exercises demonstrated on a patient with Rigo 3C curve type. **(A)** Sail exercise, **(B)** Short semihanging position, **(C)** Big bow maneuver, **(D)** Shoulder counter traction in side-lying position, **(E)** Shoulder counter traction with elastic band resistance.

The five principles of the Schroth method are autoelongation, deflection, derotation, rotational breathing, and stabilization. In this study, each exercise was individualized based on the curve type of patients and consisted of 50 breaths. The 3D postural correction incorporated movements of translation, rotation and mixed (sagittal expansions), with patients maintaining the this corrected posture even during the relaxation phase (15). Schroth exercises were specifically designed for thoracic and lumbar scoliosis curves. There are five key exercises included in the major lumber groups: muscle cylinder in standing to open lumbar concavity (Figure 2A), short semihanging for caudal-cranial spine lengthening (Figure 2B), big bow for lengthening the spine and expanding the weak side (Figure 2C), basic correction while standing between two poles (Figure 2D), and lumbar curve extension by psoassynergy activation in less stable positions (Figure 2E). For the major thoracic group, the exercises involved: standing sail exercise to stretch the thoracic concavity (Figure 3A), short semihanging for caudal-cranial spine lengthening (Figure 3B), big bow for spine lengthening and weak side expansion (Figure 2C), shoulder counter traction in side position (Figure 3D), and shoulder counter traction with elastic band for thoracic curvature correction (Figure 3E). The Schroth exercise program progressed from more to less passive support, advancing from lying to sitting and standing positions.

For participants meeting Scoliosis Research Society bracing criteria, Cheneau bracing was prescribed before the PSSE program. Following a 2-week brace adaptation period, an initial in-brace angle was measured using x-ray imaging taken after the participant had worn the brace more than 2 h by a spine surgeon blinded to this study. Patients in the part-time brace and Schroth exercise group were required to wear the brace for 14–18 h/day. Participants wore their braces mainly during nighttime and portions of the day, with a minimum daily wearing time of 14 h. The braces can be taken off when they participate in physical activities at school. In the control group, referred as the full-time brace group, received orthotic treatment, with wearing the orthotic for 20–24 h/day. All the participants were evaluated by the same internationally certified orthotist for orthotic fitting. To monitor brace compliance, a standardized log sheet was utilized under parental supervision, with weekly verification through signed documentation, and regular skin assessment at brace pressure points by the orthotist.

### Outcome measurements

Demographic information was recorded, including gender, age (years), weight (kg), height (m), body mass index (BMI, kg/m^2^), Risser sign, initial correction rate of braces, curve pattern, Cobb angle (degrees), trunk rotation angle (degrees), thoracic expansion difference, SRS-22 (subitems), and satisfaction of the participants.

#### Primary outcome

##### Cobb angle

Standard coronal whole spine standing radiographs were performed at baseline, 6 months, and 12 months of treatment. Participants were radiographed at least 24 h after removal of the brace. Cobb angle was measured from the anterior–posterior x-rays by the same PT (>5 years of experience in conservative scoliosis therapy). The angle was calculated between the upper and lower most-tilted end vertebra. The Cobb angle served as the primary indicator of AIS severity, and was significantly altered when the change was greater than 5° (15). A decrease in cobb angle more than 5° is considered as a significant improvement, an increase more than 5° is considered as deterioration, and change less than 5° is considered as stable (15, 16).

#### Secondary outcomes

##### Trunk rotation angle (ATR)

To perform the Adam test, the subject stands with feet 30 cm apart and hands together, with the PT sitting behind them. The subject bends forward slowly until the spinal deformity becomes the most prominent and the curve apex is parallel to the PT's line of sight. A scoliometer (Mizuho OSI) is placed across the apical spinous process to measure the ATR angle (17). The major ATR was measured and recorded by the same PT before and after the intervention. A significant change was produced when the ATR change was greater than 4°.

##### Thoracic expansion

The participant stands upright, and the tester measures the difference in chest circumference between deep inspiration and deep expiration at the level of the 4th intercostal space using a graduated soft tape. A difference less than 2.5 cm is regarded as abnormal. The difference in thoracic expansion is a simple indicator of cardiopulmonary function.

### Scoliosis research society 22-item (SRS-22)

The SRS-22 scale was completed by the participants in a quiet environment. The SRS-22 scale provides information on five dimensions: pain, function, self-image, mental health, and treatment satisfaction through 22 questions, with a maximum score of 5 and a minimum score of 1 for each question. The score of each dimension is the sum or average of the scores of the corresponding questions, which allows for a comprehensive assessment of the subjective perception of their condition and treatment outcomes (14).

### Statistical analysis

Statistical analysis was performed using SPSS 26.0 software. Data normality was assessed using the Shapiro–Wilk (S-W) test and homogeneity of variance was confirmed by the Levene test. A two-way repeated measures ANOVA was used to assess interaction and main effects of time and group. If the assumption of sphericity was violated, the Greenhouse-Geisser correction was applied. In the case of a significant interaction or main effect, *post-hoc* analyses were conducted with Bonferroni correction for multiple comparisons. The effects of the interventions were compared using independent samples *t*-tests for continuous variables, and paired *t*-tests were used to test for changes from baseline to post-treatment indices within each group. Data are presented as mean ± standard deviation (*x¯* ± *s*). Statistically significant was set as *P* < 0.05.

## Results

### Baseline conditions of the included patients

No significant differences were observed between the control and experimental groups in demographics or scoliosis-related indicators at baseline (Table 1). The control and experimental groups were comparable in age (*P* = 0.266), BMI (*P* = 0.825), curve pattern (*P* = 0.820), main curve Cobb angle (*P* = 0.507), ATR (*P* = 0.094), Initial in-brace correction rate (%) (*P* = 0.656), thoracic expansion (*P* = 0.552), pain (*P* = 0.312), self-image (*P* = 0.446), functional status (*P* = 0.752), and mental health scores (*P* = 0.647). In summary, patients in experiment group performed the Schroth exercise for mean 4.9 h per week and wore the brace for 14.72 h per day. Patients in the full-time group wore the brace for an average time of 21.22 h per day (Table 2).

**Table 1:** Demographic characteristics.

Characteristic	Full-time brace group	Combined treatment group	*T*	*P*
	*N* = 22 (mean ± SD)	*N* = 25 (mean ± SD)		
Age (years)	13.75 ± 1.02 (10.80–14.50)	13.46 ± 0.93 (11.00–14.50)	1.027	0.310
Weight (kg)	47.39 ± 7.93 (33.7–55.5)	48.00 ± 5.95 (40–60)	−0.302	0.764
Height (m)	1.60 ± 0.06 (1.43–1.73)	1.60 ± 0.05 (1.50–1.70)	−0.310	0.758
BMI	18.42 ± 2.65 (14.47–26.16)	18.58 ± 2.25 (14.69–23.44)	−0.223	0.824
Risser sign	1.63 ± 1.00 (0–3)	1.52 ± 0.87 (0–3)	0.426	0.672
Curve pattern (single/double)	5/17	5/20	0.052	0.820
Initial in-brace correction rate (%)	65.67 ± 8.64 (50.00–83.00)	64.08 ± 9.17 (51.00–87.00)	0.600	0.552
Cobb angle (degree)	31.63 ± 8.66 (25–40)	34.16 ± 10.28 (25–40)	−0.903	0.372
ATR (degree)	8.40 ± 2.80 (4.00–13.50)	9.96 ± 3.36 (5.00–18.00）	−1.712	0.094
Thoracic expansion (cm)	3.49 ± 0.95 (0.90–5.10)	3.60 ± 0.52 (2.50–4.50）	−0.513	0.611
Pain	4.61 ± 0.32 (3.80–5.00)	4.49 ± 0.47 (3.80–5.00)	1.022	0.312
Self-image	3.40 ± 0.52 (1.80–4.40)	3.53 ± 0.63 (2.00–4.20)	−0.768	0.446
Function	4.58 ± 0.30 (4.20–5.00)	4.54 ± 0.48 (3.40–5.00)	0.317	0.752
Mental health	3.85 ± 0.54 (2.60–4.80)	3.77 ± 0.61 (2.60–5.00)	0.460	0.647

**Table 2:** Brace wearing and Schroth exercise duration.

Characteristic	Full-time brace group (mean ± SD)	Combined treatment group (mean ± SD)
Brace wearing (h/day)	21.22 ± 1.15 (20.00–23.00)	14.72 ± 0.85 (14.00–17.00)
Schroth intervention (h/week)	0.00	4.90 ± 0.50 (4.00–6.00)

### Primary outcome

#### Cobb angle

**Figure 4:**
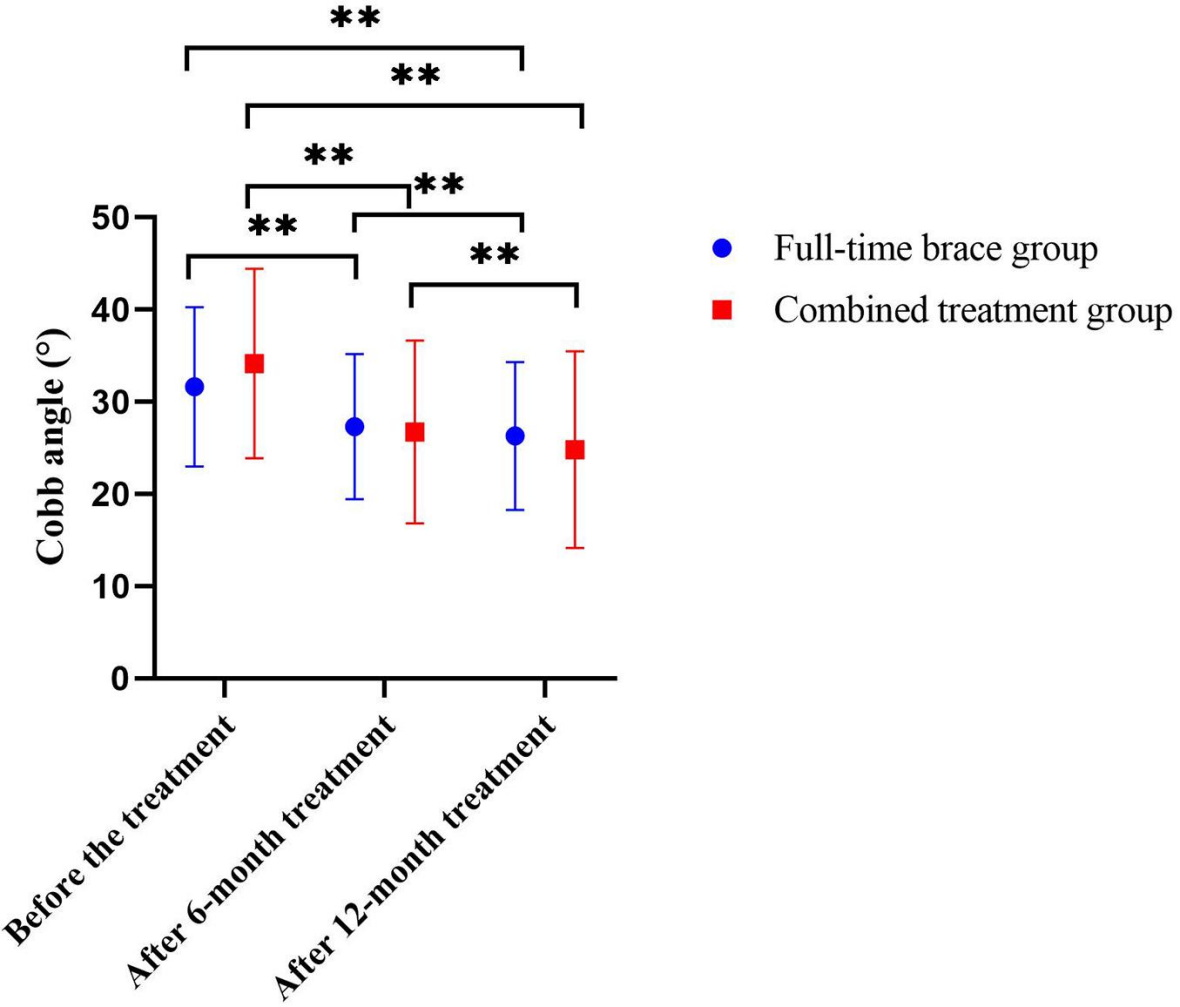
Changes in Cobb angle across three time points (baseline, 6 months, and 12 months post-treatment) for both groups. **P < 0.001.

The two-way repeated-measures ANOVA revealed a significant Time × Group interaction effect for the Cobb angle (*F* = 12.211, *P* < 0.001), indicating different rates of change between groups. Paired *t*-tests confirmed that within each group, the Cobb angle significantly decreased from baseline to both the 6 and 12-month assessments (*P* < 0.001). More importantly, independent samples *t*-tests on the change scores (post-treatment minus baseline) showed that the improvement in the experimental group was significantly greater than that in the control group at both the 6-month (*t* = −3.750, *P* < 0.001) and 12-month follow-ups (*t* = −3.614, *P* < 0.001) (see Tables 3, 4, Figure 4).

**Table 3:** Intragroup comparison before the intervention and at two follow-up time points in Cobb angles.

Outcomes	T1	T2	T3	Time × group		Partial *η*^2^	Pairwise comparison
				*F*	*P*		
Full-time brace group	31.63 ± 8.66	27.31 ± 7.87	26.31 ± 8.02	12.211	<0.001	0.213	T1–T2**; T1–T3**; T2–T3*
Combined treatment group	34.16 ± 10.28	26.72 ± 9.92	24.80 ± 10.66				T1–T2**; T1–T3**; T2–T3**;

T1, before the treatment; T2, after 6-month treatment; T3, after 12-month treatment.

* *P* < 0.05.

** *P* < 0.01.

**Table 4:** Intragroup comparison in Cobb angle at two follow-up time points.

Characteristic	Full-time brace group (mean ± SD)	Combined treatment group (mean ± SD)	*T*	*P*
After 6-month treatment	4.31 ± 2.91	7.44 ± 2.78	−3.750	<0.001**
After 12-month treatment	5.31 ± 3.53	9.36 ± 4.06	−3.614	<0.001**

** *P* < 0.001.

### Secondary outcomes

#### Comparison of the treatment efficacy

At 6-month follow-up, the combined treatment group showed greater improvements than the full-time brace group in ATR (*P* < 0.001), thoracic expansion (*P* < 0.001), pain (*P* = 0.005), and mental health scores (*P* = 0.047) on the SRS-22. However, there was no significant difference between the two groups in terms of functional status (*P* = 0.161) and self-image (*P* = 0.591) (Table 5).

**Table 5:** Intergroup comparison before and after 6-month follow-up.

Characteristic	Full-time brace group (mean ± SD)	Combined treatment group (mean ± SD)	*T*	*P*
ATR (degree)	0.70 ± 1.10	2.94 ± 1.01	−7.248	<0.001**
Thoracic expansion (cm)	0.13 ± 0.87	2.35 ± 0.84	−8.785	<0.001**
Pain	−0.08 ± 0.23	0.10 ± 0.19	−2.969	0.005*
Self-image	0.10 ± 0.45	0.04 ± 0.29	0.542	0.591
Function	−0.01 ± 0.15	0.07 ± 0.22	−1.424	0.161
Mental health	0.14 ± 0.47	0.38 ± 0.33	−2.044	0.047*

* *P* < 0.05.

** *P* < 0.001.

#### Efficacy of Schroth exercise with part-time brace therapy

In the Schroth 3D exercise with the part-time brace group, there were significant improvements in ATR (*P* < 0.001), thoracic expansion (*P* < 0.001), pain (*P* = 0.012), and mental health (*P* < 0.001) after 6 months of treatment compared to baseline. There were no statistically significant differences in self-image (*P* = 0.503) and function (*P* = 0.119) (Table 6).

**Table 6:** Intragroup comparison of continuous variables for Schroth 3D exercise with part-time brace.

Characteristic	Before the treatment (mean ± SD)	After 6-month treatment (mean ± SD)	*T*	*P*
ATR (degree)	9.96 ± 3.36	7.02 ± 3.45	14.553	<0.001**
Thoracic expansion (cm)	3.60 ± 0.52	5.95 ± 0.73	−13.321	<0.001**
Pain	4.49 ± 0.47	4.60 ± 0.43	−2.701	0.012*
Self-image	3.95 ± 0.67	4.16 ± 0.66	−0.679	0.503
Function	4.54 ± 0.48	4.61 ± 0.37	−1.616	0.119
Mental health	3.77 ± 0.61	4.16 ± 0.66	−5.710	<0.001**

* *P* < 0.05.

** *P* < 0.001.

#### Efficacy of full-time brace treatment

In the full-time brace group, significant improvements from the baseline to 6-month follow-up were observed in ATR (*P* = 0.007). However, there were no statistically significant differences in the other clinical assessments such as thoracic expansion (*P* = 0.475), pain (*P* = 0.119), function (*P* = 0.789), self-image (*P* = 0.316) and mental health in SRS-22 questionnaire (*P* = 0.178) (Table 7).

**Table 7:** Intragroup comparison of continuous variables in the full-time brace group.

Characteristic	Before the treatment (mean ± SD)	After 6-month treatment (mean ± SD)	*t* value	*p-*value
ATR (degree)	8.40 ± 2.80	7.70 ± 2.88	2.980	0.007*
Thoracic expansion (cm)	3.49 ± 0.95	3.62 ± 0.82	−0.727	0.475
Pain	4.61 ± 0.32	4.53 ± 0.38	1.624	0.119
Self-image	3.40 ± 0.52	3.50 ± 0.67	−1.027	0.316
Function	4.58 ± 0.30	4.57 ± 0.33	0.271	0.789
Mental health	3.85 ± 0.54	3.99 ± 0.60	−1.392	0.178

* *P* < 0.05.

#### Comparison of patient satisfaction between the two groups

At 6-month follow-up, patient satisfaction was comparable in both groups (*P* = 0.576) (Table 8).

**Table 8:** Intergroup comparison of patient satisfaction.

Characteristic	Full-time brace group	Combined treatment group	*t* value	*p*-value
Satisfaction	4.04 ± 0.72	4.16 ± 0.67	−0.563	0.576

## Discussion

This randomized controlled study showed that both Schroth correction training combined with a 14–18 h/day brace and full-time 20–24 h/day brace treatment had a beneficial effect on Cobb's angle during 6 months of therapy and a 12-month follow-up for patients with AIS (*P* < 0.01). However, the part-time brace combined with Schroth exercise group generated greater improvements than the full-time brace treatment group (*P* < 0.01). For secondary outcomes, the experimental group showed superior improvements in ATR (*P* < 0.05), thoracic expansion (*P* < 0.05), and the pain and mental health domains of the SRS-22 questionnaire (*P* < 0.05) compared to controls at 6 months. A potential mechanism is that physical exercise enhances the endurance of paravertebral musculature, thereby promoting spinal stability through improved neuromuscular control and structural support (18). The Schroth exercise is a therapeutic approach that combines cognitive, sensory-motor, and kinesthetic training to improve 3D posture in patients with scoliosis. This method is founded on the “vicious cycle” model, which posits that scoliotic posture promotes curve progression (19). The daily postural management and conscious maintenance of alignment emphasized in the Schroth method work to reduce asymmetric spinal loading, thereby potentially slowing curve progression and alleviating pain. Since our cohort predominantly consisted of patients at Risser stage II, a phase of reduced efficacy for purely growth-guided bracing, the corrective principles must adapt by placing greater emphasis on axial muscle strengthening to secure and stabilize the correction. As patients progress in skeletal maturity throughout the intervention period, the physiological basis for growth modulation diminishes, which could account for the observed tapering of the corrective rate in the later follow-ups. Yu et al. (20) conducted a comparative study to evaluate exercise therapy verus 23-hour bracing, they found that both groups improved the scoliosis parameters. However, superior quality of life outcomes was observed in the exercise group. Consistent with these findings, our study showed that bracing combined with exercise led to greater improvements in pain and psychological well-being compared to bracing alone. The observed changes may partly be attributed to the regular contact with healthcare professionals, which enhanced patient compliance.

Our study demonstrated that combining Schroth training with part-time bracing led to superior improvement in the Cobb angle compared to the full-time bracing group. The primary challenge in AIS conservative management lies in optimizing the trade-off between therapeutic efficacy and minimizing the impact on the adolescent's physical and psychosocial development. Full-time bracing with rigid orthoses like the Cheneau brace remains a cornerstone of treatment due to its well-documented efficacy in curve correction (21). However, the requirement for near-constant wear can pose challenges to physical activity, comfort, and psychological well-being for some patients, as noted in some studies (9, 22). Conversely, other studies have shown that with proper design and patient support, bracing can be associated with acceptable tolerance and quality of life (23). Parallelly, the Schroth method offers a proactive approach by aiming to improve muscular symmetry, postural control, and cardiopulmonary function (24, 25), though their standalone corrective power for larger curves may be limited. Integrating Schroth exercises with part-time bracing might harness a synergistic effect: the brace provides essential mechanical correction, while the exercises promote active spinal stabilization and address functional limitations. Our results support this premise. These findings suggest that such an integrated regimen represents a viable and potentially preferable clinical option, particularly for patients seeking a less restrictive treatment schedule. It may mitigate some burdens associated with prolonged brace wear while maintaining correction through active patient engagement.

In our study, braces combined with Schroth exercise were more effective in reducing ATR compared to braces alone. The severity of spinal rotation correlates directly with cosmetic deformity. Highly rotated spines are associated with razorback deformity, thoracic deformity, and transverse trunk displacement, and compromised cardiopulmonary function. ATR measurements serve as a key indicator for monitoring idiopathic scoliosis progression. Our findings align with previous research demonstrating that Schroth training can significantly reduce ATR (26). For example, the participants in one study who underwent 10 weeks of Schroth training showed significant improvements in thoracic trunk rotation angle, and cosmetic trunk deformity (1). A 30-month prospective cohort study of 55 patients using Gensingen's brace, which revealed statistically significant improvement in ATR (27).

Our study investigated the effects of bracing and exercise on thoracic expansion differences, a simple index for pulmonary function assessment. The thoracic expansion is used to identify whether bracing restricts thoracic expansion, and whether exercise training can mitigate potential negative effects. Gozde et al. (28) conducted a 1-month study of 27 adolescent AIS patients, measuring pulmonary function with or without brace wear. They found that bracing results in reduced spirometry values, decreased exertional expiratory volume, diminished exertional spirometry, lower maximum ventilatory capacity, and decreased peak expiratory flow values, and increased dyspnea. Scoliosis patients present with bilateral asymmetry in thoracic motion, abnormal thoracic movement during respiratory exercise, and reduced mobility of the lower lung border. These factors limit thoracic movement and result in reduced thoracic volume. Our findings demonstrated no significant change in thoracic mobility in patients using bracing alone. Nevertheless, patients with a brace combined with Schroth correction training showed a significant improvement in thoracic mobility compared full-time brace group after 6 months. An 8-month follow-up investigation by Yasin et al. (29) of 15 brace-treated AIS patients found a transient limiting effect of the brace on pulmonary function. No permanent lung function changes were found after 8 months. Kim et al. (30) found that 12 weeks of Schroth exercise significantly improved Cobb angle and spirometry value.

We found a statistically significant improvement in both pain and psychological well-being in patients with combined brace and exercise therapy compared to those using braces alone. Research indicates that optimal brace treatment protocols should address three key factors affecting patients’ quality of life: self-image, psychological well-being, and vitality. Treatment compliance is associated with the patient initiative, psychological factors, and pain levels. Curve progression and worse treatment outcomes often result in poor compliance. Back pain and psychological well-being significant impact patients’ quality of life (9). Research indicates that patient compliance rates vary by age: 72% among 10–12 years old, 49% for ages 12–14, and 47% for ages 14–16, with younger patients showing higher adherence (31). Deniz et al. (10) conducted a 7-day Schroth boot camp for 45 adolescents with AIS, with 4.5 h of daily corrective training. They found improved postural symmetry and significant improvements in aesthetic and health-related quality of life. While literature indicates that full-time bracing can negatively impact patients’ quality of life across psychological and social domains (32), its biomechanical rationale for curve correction remains a cornerstone of treatment. The initial phase of full-time bracing may function by inducing visco-plastic creep in the concave tissues, thereby establishing a structural foundation that enhances the effect of concurrent physical therapy. This supports the logic of a sequential dosing strategy, specifically an initial period of full-time wear to maximize correction, followed by a transition to part-time bracing. Such a phased approach seeks to balance biomechanical efficacy with the need to mitigate psychosocial burdens, aligning with the broader call for an integrated, interdisciplinary model of care.

The limitations of the study and future directions should be pointed out. Firstly, objective monitoring via smart sensors (e.g., pressure/temperature) integrated into braces is needed. A critical design principle is to ensure such technology does not discourage physical activity. Sensors should therefore be capable of differentiating legitimate removal periods (as during sports) from non-compliance, preserving both data integrity and patient well-being. In addition, follow-up assessments should be extended until skeletal maturity is reached to better evaluate the long-term impact of the intervention on spinal curvature and overall skeletal development (33). Also, future research should include additional outcome measures beyond the Cobb angle in follow-up assessments to provide more solid evidence of treatment efficacy (34). Finally, it should be acknowledged that the Schroth-based protocol in this study required a considerable time commitment of approximately 5 h per week. This intensity may challenge long-term adherence and could potentially limit patients’ participation in other valued activities, such as organized sports (35). Future research could explore more time-efficient models or establish dose-response relationships.

## Conclusion

Our study provides short-term evidence supporting the efficacy of a combined part-time bracing and intensive Schroth exercise protocol. The results suggest that within a 12-month period, this regimen can yield superior outcomes compared to full-time bracing alone, positioning Schroth therapy as a crucial active complement to passive bracing. This indicates its potential as a more tolerable and patient-friendly treatment option in the intermediate term.

## Data Availability

The datasets presented in this study can be found in online repositories. The names of the repository/repositories and accession number(s) can be found below: https://doi.org/10.6084/m9.figshare.30932621.
